# Severe pulmonary hypertension in weaning from cardiopulmonary bypass following double Ozaki procedure: a case report

**DOI:** 10.1186/s40981-025-00785-w

**Published:** 2025-04-21

**Authors:** Jin Sato, Hideki Hino, Ryota Watanabe, Takashi Mori

**Affiliations:** Department of Anesthesiology, Osaka Metropolitan University Graduate School of Medicine, Osaka, Japan

**Keywords:** Pulmonary hypertension, Vasopressin, Ozaki surgery

## Abstract

**Background:**

Ozaki surgery, which reconstructs cardiac valves using autologous pericardium, is commonly performed for aortic valve replacement and offers benefits such as avoiding anticoagulation and reducing valve degeneration. However, its application to pulmonary valve replacement remains rare, and anesthetic management for such cases is not well documented.

**Case presentation:**

A 71-year-old woman with severe aortic and pulmonary valve stenosis underwent double valve replacement using the Ozaki procedure and coronary artery bypass grafting. Post-cardiopulmonary bypass, she developed severe pulmonary hypertension and systemic hypotension. Norepinephrine exacerbated pulmonary hypertension, while arginine vasopressin effectively stabilized systemic pressure without worsening pulmonary pressure.

**Conclusions:**

This is the first reported case of anesthetic management for double valve replacement using the Ozaki procedure. Adequate use of vasopressin led to ameliorating pulmonary hypertension after cardiopulmonary bypass. Assessing preoperative right ventricular pressure and selecting appropriate vasopressors are crucial in mitigating perioperative pulmonary hypertension.

## Background

Currently, biological tissues and mechanical valves are mainly used for cardiac valve replacement surgery. However, patients with a mechanical valve (MV) require the internal use of warfarin throughout their lives because of the risk of thromboembolism. On the other hand, biological tissue valves (BVs) do not require anticoagulants as long as mechanical valves do, but due to degeneration caused by chronic autoimmune inflammatory responses, younger patients are at risk of requiring repeat cardiac valve replacement in the future [1]. The method of valve repair using autologous pericardium was first reported by Duran et al. [2]. Subsequently, Ozaki et al. devised a new procedure using separate pericardial leaflets for each coronary cusp, called the Ozaki procedure [3]. This technique is more physiological for cardiac hemodynamics than the use of MVs and BVs, which leads to the avoidance of valve calcification due to autoimmune inflammation and the risk of bleeding due to anticoagulation [4, 5]. However, there have been few reports of valve replacement other than aortic valve replacement using the Ozaki procedure and few reviews of intraoperative complications and management in such a surgery. In this case report, we describe the anesthetic management and perioperative progress of patients who underwent double valve replacement for the aortic and pulmonary valves using the Ozaki procedure. The patient provided written consent for the publication of this case report.

## Case presentation

A 71-year-old woman (142 cm, 42 kg) with aortic valve stenosis, pulmonary valve stenosis, and angina pectoris underwent aortic valve plasty, pulmonary valve plasty with an autologous pericardium (Ozaki procedure), and coronary artery bypass surgery. Her medical history included bronchial asthma, hypertension, and type II diabetes mellitus. She developed a cerebral infarction at the age of 60 years without sequelae. The patient’s preoperative status was New York Heart Association Class II. A preoperative transthoracic echocardiogram showed a left ventricular ejection fraction of 61%, tricuspid annular plane systolic excursion of 12 mm, severe aortic stenosis with valve area of 0.47 cm^2^, mean peak gradient of 83 mmHg. Severe pulmonary valve stenosis with maximum velocity of 5 m/s and tricuspid regurgitation with peak gradient of 130 mmHg were also shown. Preoperative catheter examination revealed a pulmonary artery pressure of 26/12/19 mmHg (systolic/diastolic/mean), Right ventricular pressure of 167/7 mmHg, right atrial pressure of 13/4/4 mmHg, and aortic pressure of 158/86/16 mmHg. Blood test results were unremarkable except for N-terminal pro-brain natriuretic peptide of 2756 pg/ml.

With monitoring of arterial blood pressure and electroencephalogram besides standard monitoring, general anesthesia was induced with propofol 60 mg, remifentanil 0.4 µg/kg/min, rocuronium 40 mg, and maintained with sevoflurane 1.0–1.5% with remifentanil 0.15–0.3 µg/kg/min. A pulmonary artery catheter inserted via the right jugular vein was not advanced to the pulmonary artery due to pulmonary valve stenosis and was placed in the right ventricle. The hemodynamic parameters were stable with systolic arterial blood pressure (SAP) of 120 mmHg; central venous pressure (CVP) of 6 mmHg, right intraventricular pressure of 79/5/24 mmHg measured by pulmonary artery catheter, although right intraventricular pressure occasionally exceeded systemic blood pressure.

The surgery was performed as planned preoperatively. First, the aortic valve was repaired with autologous pericardium. Next, the pulmonary valve was also repaired using the Ozaki procedure. The operator removed damaged leaflets and measured intercommissural distance using a sizer. After that, he cut the pericardium on the template. The aortic valve was repaired with autologous pericardium left coronary cusp 21 mm, right coronary cusp 23 mm and non-coronary cusp 23 mm, which formed by using Ozaki template. All three cusps of the pulmonary artery valve were formed 24 mm by the template. Cardiopulmonary bypass was finished 427 min after the start of surgery. The outlet of the pulmonary artery was enlarged, and coronary artery bypass grafting was performed. Before completion of the surgery, the pulmonary artery catheter was advanced into the pulmonary artery by the surgeon.

**Fig. 1:**
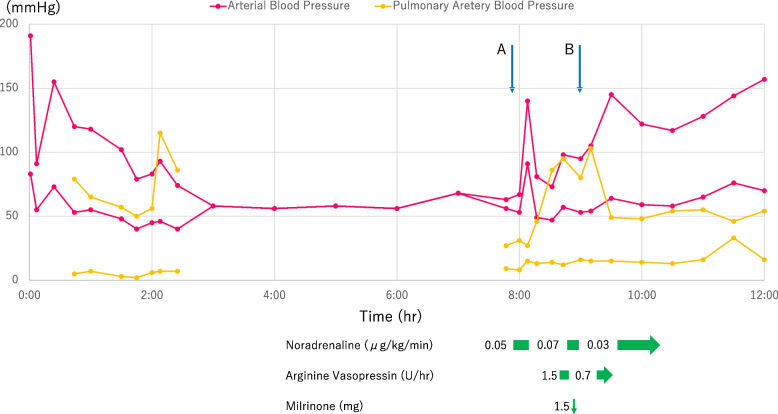
Time courses of systemic arterial pressure and pulmonary arterial pressure in double-Ozaki surgery. The horizontal axis is the time from the start of anesthesia. **A** indicates the end of cardiopulmonary bypass. **B** indicates when the bolus of milrinone and the starting continuous of arginine vasopressin (AVP) was administrated

Upon the weaning of cardiopulmonary bypass, continuous infusion of dopamine 5 µg/kg/min and norepinephrine 0.05 µg/kg/min were initiated. Systemic hypotension persisted despite an increased continuous infusion dose and bolus of norepinephrine. Systemic arterial blood pressure was 99 mmHg; CVP, 16 mmHg; and PAP increased to 98/15/44 mmHg. Bolus milrinone was not effective for decreasing PAP, and norepinephrine was replaced with arginine vasopressin (AVP) 0.2 units bolus repeatedly, followed by continuous infusion at 1.6 units/h, which effectively ameliorated pulmonary hypertension The AVP dose was reduced to 0.7 units/h and totally, administered for 40 min. The total dose was 0.9 units. Systemic arterial pressure was maintained at approximately 100 mmHg, whereas systemic PAP decreased to 44 mmHg. The PaO_2_/FiO_2_ ratio remained > 300 throughout the post-CPB period. The surgery time was 639 min. The patient was then transferred to the intensive care unit under sedation and intubated. Systolic PAP was approximately 40 mmHg until the day after surgery (Fig. 1). After admission to the ICU, with administered dopamine and dobutamine continuously, mild pulmonary hypertension (systolic PAP was 44 mmHg) remained, but hemodynamics was maintained (Cardiac Index was 2.3 L/min/m^2^). The tracheal tube was removed on the first postoperative day. X-rays showed phrenic nerve paralysis, but oxygenation was maintained despite a slight increase in PaCO2. Later, aspartate transaminase (AST) and alanine transaminase (ALT) increased, and right heart failure was suspected, so a transthoracic echocardiogram was performed on postoperative day 10. Then, we revealed a hematoma around the right atrium, and pericardial drainage was performed under local anesthesia. Thereafter, the patient’s condition improved, and he was discharged from the ICU on postoperative day 13.

## Discussion

Ozaki procedure was originally reported as aortic valve reconstruction using an autologous pericardium [3], In addition to a lack of requirement of postoperative anticoagulants and low incidence of reoperation within the midterm follow-up period with an average of 54 months [6], it has been applied to the repair of the pulmonary valves later [7]. Severe aortic stenosis and pulmonary stenosis with an old patient is very rare and we found only one case report of biological valve replacement with them [8]. In the case report, the patient had an uneventful postoperative course and hemodynamics. Because intraoperative hemodynamic conditions during Ozaki procedure is more stable than aortic valve replacement surgery [4], there are few special precautions to be taken in anesthetic management other than the assessment of valve function upon weaning from cardiopulmonary bypass.

The autologous pericardium has preferable characteristics such as a normal valve, providing a large effective orifice area and a very low transvalvular pressure gradient, while artificial valves lead to a transvalvular pressure gradient and strict blood flow from the right ventricle. This may worsen pulmonary hypertension due to the large output from the hypertrophic right ventricle. In our case, impaired cardiac contractility after cardiopulmonary bypass in addition to preexisting high right intraventricular pressure and pulmonary hypertension may have led to low cardiac output and systemic hypotension.

The patient was administered norepinephrine during weaning from cardiopulmonary bypass. Although systemic hypotension was slightly ameliorated, pulmonary hypertension worsened by increasing norepinephrine dose. Arginine vasopressin is one of the most suitable vasopressors for this condition by increasing systemic blood pressure without increasing PAP [9]. Also, in the study using human tissue, AVP constricted the radial artery but not the pulmonary artery [10].

Milrinone effectively reduces pulmonary resistance and is used to treat pulmonary hypertension. However, systemic resistance was also reduced, resulting in hypotension. Arginine vasopressin is a suitable counter-vasoactive agent for SVR reduction using milrinone as shown in off-pump coronary artery bypass graft surgery [11]. Nitric oxide was not selected in this case because of a lack of hypoxia and complicated systems for its inhalation. One limitation is that detailed information of cardiac function, such as transesophageal echocardiography, or cardiac index which could be obtained using a pulmonary artery catheter was not available, making the effect of milrinone administration on pulmonary artery and cardiac function unclear.

In conclusion, this paper is the first case report on anesthesia management in an Ozaki procedure performed on two valves. The patient developed pulmonary hypertension after weaning from cardiopulmonary bypass but recovered with appropriate management. In Ozaki procedure for the pulmonary valve, it may be important to assess the risk of pulmonary hypertension by evaluating right ventricular pressure and to select an appropriate vasopressor.

## Data Availability

Not applicable.

## Abbreviations

AVP: Arginine vasopressin
SAP: Systolic arterial blood pressure
CVP: Central venous pressure
PAP: Pulmonary arterial pressure
